# Mechanism of sea-ice expansion in the Indian Ocean sector of Antarctica: Insights from satellite observation and model reanalysis

**DOI:** 10.1371/journal.pone.0203222

**Published:** 2018-10-03

**Authors:** Babula Jena, Avinash Kumar, M. Ravichandran, Stefan Kern

**Affiliations:** 1 ESSO - National Centre for Antarctic and Ocean Research, Ministry of Earth Science, Government of India, Vasco-da-Gama, India; 2 Integrated Climate Data Center (ICDC), Center for Earth System Research and Sustainability, University of Hamburg, Hamburg, Germany; Universidade de Aveiro, PORTUGAL

## Abstract

In the backdrop of global warming, Antarctic sea-ice variability showed an overall expansion with the regional heterogeneity of increasing and decreasing patterns. Analysis of satellite derived sea-ice extent, during 1979 to 2015, in the Indian Ocean sector of Antarctica (IOA) revealed expansion of 2.4±1.2% decade^-1^. We find strengthening of westerly wind during the austral summer (between 50°S to 62°S) facilitated northward advection of a cool and fresh layer. Also, the strong westerly wind cools the upper ocean due to net heat loss from the ocean surface. The combined effect of northward advection of cold fresh layer and net heat loss from the surface, favours sea-ice expansion in the subsequent seasons, in the IOA region, north of 62°S. However, sea-ice retreat was observed near the Kerguelen Plateau, due to upper ocean warming, and hence a non-annular pattern of sea-ice extent in the IOA was observed.

## Introduction

The Southern Ocean plays a crucial role in the Earth’s climate system and its sea-ice coverage is a key indicator of climate change that modulates the albedo, air-sea exchange of heat, fresh water, carbon, ocean-atmospheric circulation, Antarctic ecosystems, and biogeochemical cycle [1,2]. The influence of global warming on the Arctic sea-ice shows a profound declining trend as expected, in contrast to the Antarctic sea-ice expansion. Several authors reported a significant increase in Antarctic sea-ice extents [3–6] which has been linked to westerly wind forcing [7–11]; southern annular mode (SAM) [7,12–14]; El Niño–Southern Oscillation (ENSO) [15]; stratospheric ozone depletion [16–21]; precipitation [22]; and ocean temperature [23,24]; however, the overall sea-ice expansion masks large regional variations [25]. At present there is no consensus on the underlying mechanism to explain the observed sea-ice expansion. Regional scale processes and unidentified large-scale processes should be investigated to explain the observed sea-ice expansion [26]. The interpretations of regional sea-ice variability around Antarctica have been focussed on the most part of the Atlantic and the Pacific Ocean. Importance of the Indian Ocean sector of Antarctica is known for the bottom water formation due to enhanced sea-ice production and subsequent brine rejection [27], influencing the global climate through meridional redistribution of heat, and polar ecosystem [28]. Considering its importance, regional studies on sea-ice dynamics in the Indian Ocean Sector of Antarctica (IOA) are essential from a global weather and climate perspective [29]. In this article, we first show the satellite derived sea-ice variability in the IOA (20°E to 90°E), followed by a mechanism on the role of westerly wind and the ocean current for driving the theromhaline structure to cause the sea-ice growth are examined. Further, the observed growth in sea-ice that masks the significant regional variations (sea-ice retreat) in some places near to the coast and south Kerguelen Plateau, are investigated.

## Materials and methods

The data sets used in this study are: (i) Monthly sea-ice extent and concentration (1979–2015) from the Scanning Multichannel Microwave Radiometer (SMMR), the Special Sensor Microwave Imager (SSMI), and the Special Sensor Microwave Imager Sounder (SSMIS) in polar stereographic projection at 25×25 km spatial resolution acquired from the National Snow and Ice Data Center (NSIDC), Colorado; (ii) National Oceanic and Atmospheric Administration (NOAA) monthly optimum interpolated sea surface temperature (OI SST) v2 (1982–2015) at a spatial resolution of 1°×1° constructed after blending of Advanced Very High Resolution Radiometer (AVHRR) and *in-situ* observations [30]; (iii) Quality controlled monthly potential temperature, salinity, and current vector (1979–2015) from the European Centre for Medium-Range Weather Forecast (ECMWF)’s advanced operational ocean reanalysis system (ORAS4) and objective analysis product from Met Office Hadley Centre EN4 (EN.4.2.0) at a spatial resolution of 1°×1° with up to ~10 m vertical resolution; (iv) ECMWF’s ERA-Interim monthly reanalysis data (1979–2015) on wind vector (10 m), and total column ozone at a spatial resolution of 25×25 km; (v) SAM monthly index (1979–2015) from National Center for Atmospheric Research (NCAR) [13]; (vi) Gravity Recovery and Climate Experiment (GRACE) estimated monthly mass balance data (2002–2015) for Antarctic drainage basins from Technische Universität Dresden, Germany [31]; and (vii) Locations of various Southern Ocean climatological fronts from the Australian Antarctic Data Centre [32]. In this article, we demarcated five different regional sectors of the Southern Ocean: Weddell Sea (60°W–20°E), Indian Ocean (20°E–90°E), western Pacific Ocean (90–160°E), Ross Sea (160°E–130°W), and the combined Bellingshausen and Amundsen Seas (BAS, 130°W –60°W), following Parkinson and Cavalieri, (2012) [33].

The sea-ice extent and concentration are derived from passive microwave brightness temperature using the National Aeronautics and Space Administration (NASA) algorithm for the period of 1979–2015. The sea-ice extent is computed by summing-up the areas of all pixels having at least 15% of sea-ice concentration in the IOA between 20°E to 90°E [5]. We computed seasonal averages for sea-ice extent, sea-ice concentration, sea surface temperature (SST), wind, net heat flux, ocean current, potential temperature, and salinity from monthly data corresponding to four different austral seasons (Summer—January to March; Autumn—April to June; Winter—July to September; Spring—October to December). The seasonal cycle for each year is removed by subtracting 37-year seasonal mean values [34]. The time series of data are fitted with a linear function to model the trend by using a least square method. Similarly, the zonal average (20°E to 90°E) trends of potential temperature, salinity, and ocean current are computed for different seasons. ORAS4 is an ocean reanalysis system where in both *in-situ* and satellite data are being assimilated. EN4 is quality controlled subsurface ocean temperature and salinity profiles and objectively analysed data. The detailed methodology of product generation, and quality control approaches were given elsewhere for ORAS4 [35,36] and EN4 [37]. In order to experiment whether the sea surface cooling has occurred as an effect of heat transfer from the ocean, the net heat flux trend was computed during 1979–2011 from ECMWF’s ORAS3 [38]. Ocean current trends from ORAS4 were calculated separately for the zonal and meridional components and plotted the vector trends. Only the statistically significant trend values were considered with *p*-values less than 0.05 (at 95% confidence level) according to a two-tailed t-test. The water masses such as Antarctic surface water (AASW), Antarctic intermediate water (AAIW), and circumpolar deep water (CDW) are characterised after following the standard methodology [39,40].

In order to verify whether the observed increase in sea-ice extent and concentration are compensated by the loss of sea-ice volume, we compute the sea-ice freeboard flux, i.e., sea-ice freeboard ‘F’ times sea-ice area ‘A’ times the relevant component of the sea-ice motion vector ‘v’ for the period 2002 through 2017 for the gates denoted in S1 Fig. We use the sea-ice freeboard flux instead of the sea-ice (thickness) volume flux because the sea-ice thickness obtained from the sea-ice freeboard is less good evaluated than the sea-ice freeboard itself. In addition, it contains more uncertainties due to assumptions required for the sea-ice freeboard to thickness conversion. In particular, the usage of a snow-depth climatology challenges the interpretation of these sea-ice thickness values when using a time series as is done in this article. Sea-ice freeboard ‘F’ as computed from Envisat (until March 2012) and CryoSat-2 (since November 2010) satellite altimetry [41,42] within the ESA-CCI sea-ice ecv project (see http://esa-cci.nersc.no) is available, e.g. from the Copernicus data portal for Envisat: http://catalogue.ceda.ac.uk/uuid/b1f1ac03077b4aa784c5a413a2210bf5 and CS2: http://catalogue.ceda.ac.uk/uuid/48fc3d1e8ada405c8486ada522dae9e8. The sea-ice area ‘A’ derived from the combined Eumetsat OSI-SAF / ESA-SICCI-2 AMSR-E / AMSR2 sea-ice concentration (SIC) data set (OSI-450: http://dx.doi.org/10.15770/EUM_SAF_OSI_0008, SICCI-50km: http://dx.doi.org/10.5285/5f75fcb0c58740d99b07953797bc041e) [43]. SICCI-50km SIC data are used throughout the entire AMSR-E–AMSR2 period; OSI-450 SIC data are used to fill the gap with AMSR-E / AMSR2 observations from October 2011 through July 2012. OSI-450 SIC data, which have 25 km grid resolution, were re-sampled to the 50 km grid resolution of the SICCI-50km data set. From these daily SIC data we computed monthly averages. For sea-ice motion we use the NSIDC sea-ice motion data set v03 [44] (http://nsidc.org/forms/nsidc-0116_or.html) with monthly temporal resolution. For meridional fluxes at gates 1 and 2 we only use the v-component of the ice motion; for zonal fluxes at gates 3 to 6 we only use the u-component of the ice motion. We co-locate the sea-ice motion vectors, which are on the NSIDC polar-stereographic grid with tangential plane at 70°S, onto the EASE2.0 grid used for the SIC and sea-ice thickness data. We only use cases with SIC > 70% in accordance with the settings of the sea-ice freeboard retrieval [41]. We used the uncertainty information provided with the SIC and sea-ice thickness data sets to compute an uncertainty of our sea-ice freeboard fluxes. In S2–S4 Figs, we show time series of the total sea-ice freeboard flux at the gates shown in S1 Fig together with the uncertainty (error bars). Envisat and CS-2 based estimates are shown with different symbols. We did not carry our any inter-sensor bias correction but we point out that the sea-ice freeboard retrieval was optimised to mitigate the inter-sensor bias [41].

## Results and discussion

### Observed sea-ice expansion

**Fig 1:**
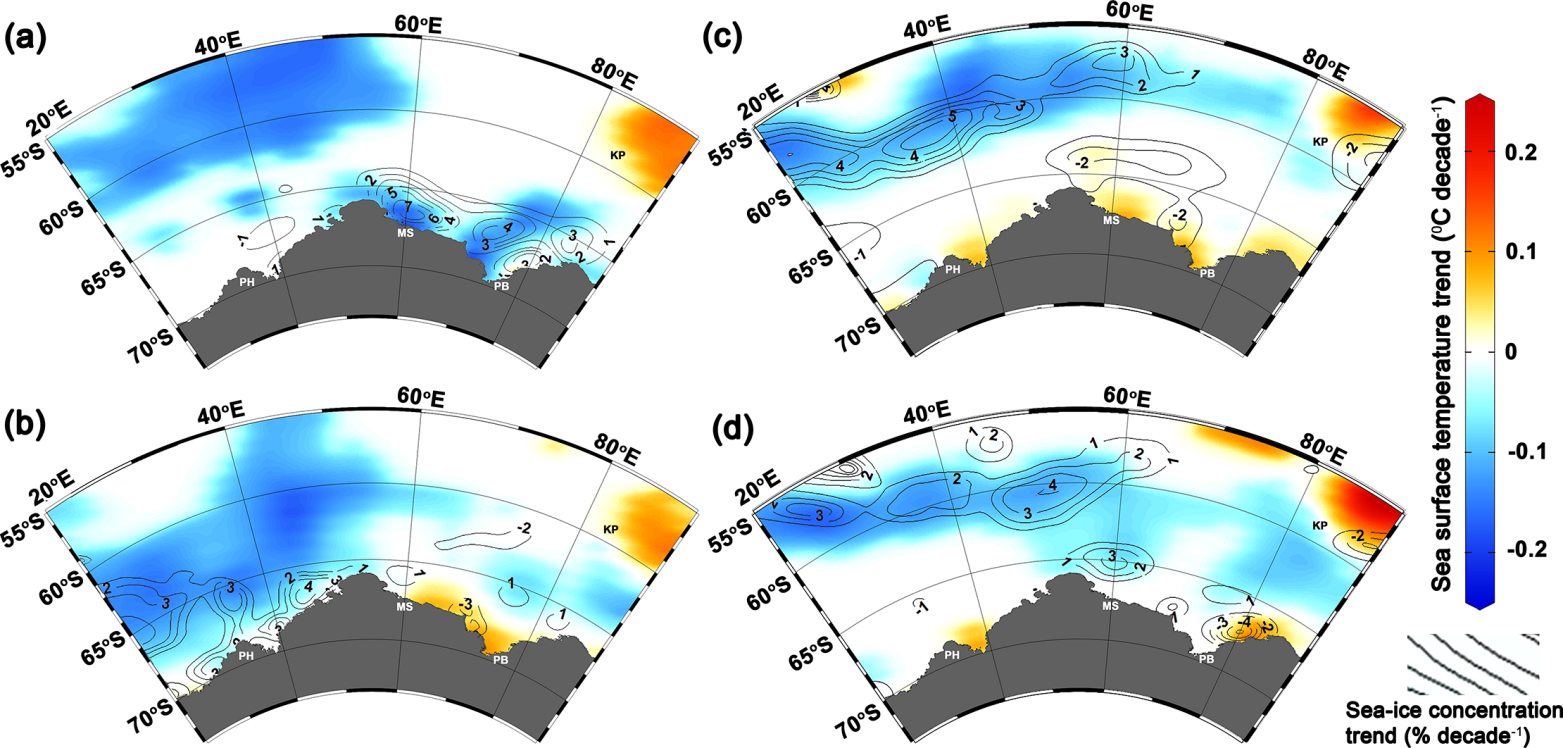
Satellite derived sea-ice concentration trend contours (% decade^-1^) overlaid on the optimum interpolated sea surface temperature trend (°C decade^-1^) at p<0.05, for (**a**) summer, (**b**) autumn, (**c**) winter, and (**d**) spring. Blue and red colours indicate sea surface cooling and warming trend respectively. Overall, the increasing pattern of sea-ice concentration is evident (associated with cooling) in the Indian Ocean sector of Antarctica (IOA) except the reduction in sea-ice near the south Kerguelen Plateau (SKP), and coastal regions of the Prydz Bay (PB), Mawson (MS), Prince Harald (PH).

**Fig 2:**
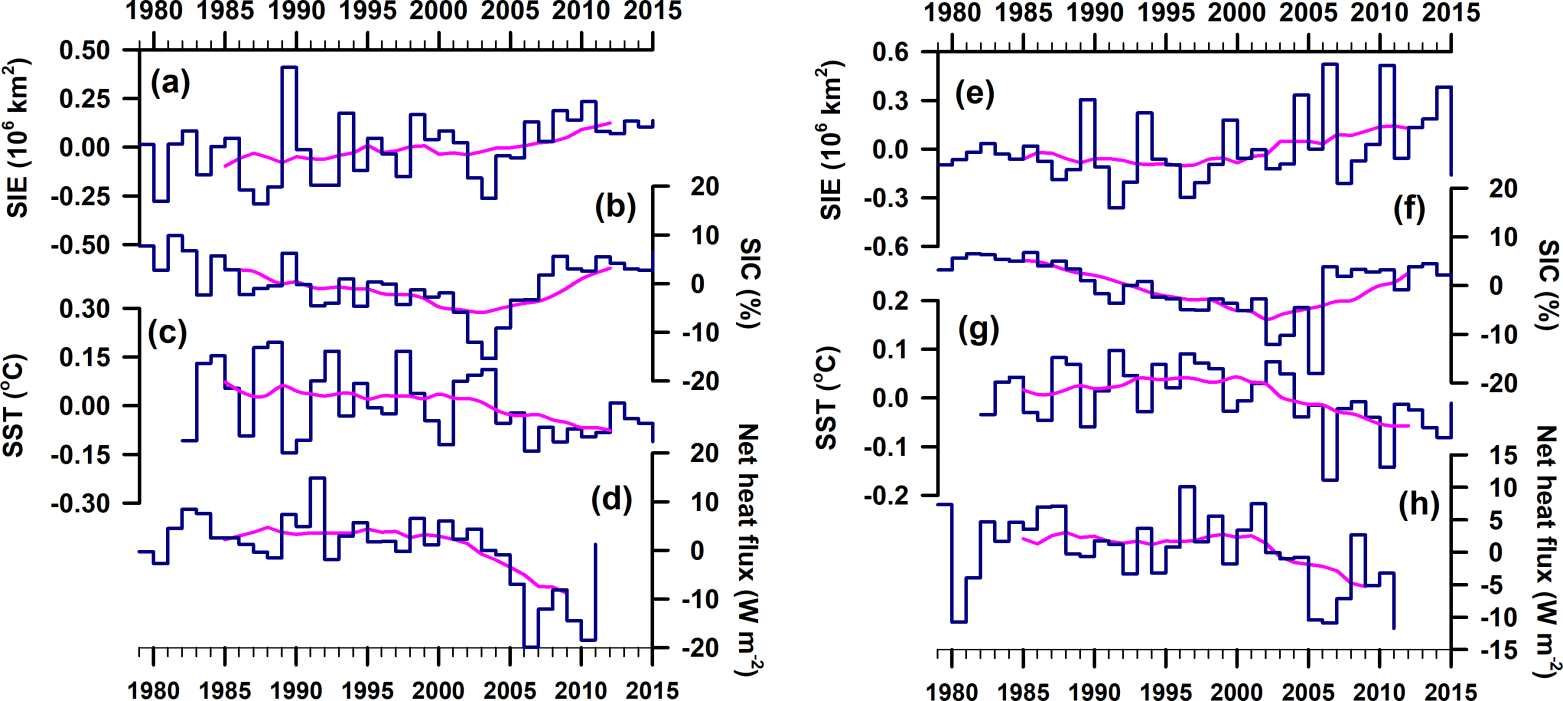
Anomalies of (**a**) sea-ice extent, (**b**) sea-ice concentration, (**c**) sea surface temperature, and (**d**) net heat flux, during the austral autumn (1979–2015) over the IOA (averaged from 20°E to 90°E and 73°S to 55°S). Right panel figures (Figs **e** to **h**) are same as that of left panel, but for the austral winter. The pink lines are the running mean of 10-year data.

In accordance with recent studies [45,46] we find an overall expansion of Antarctic sea-ice extent over the 37-year period (1979–2015) by 24.9×10^3^ ± 4.4×10^3^ km^2^ yr^-1^ (or 2.1% decade^-1^, *p* = 2.14×10^−6^) with regional heterogeneity that comprises of increasing and decreasing pattern in different sectors (Table 1). For the IOA we find a significant expansion of sea-ice extent at a rate of 2.4%±1.2 per decade. The trends in IOA sea-ice extent have a substantial seasonal variability which peaks in summer (10.6%±4.1 decade^-1^), followed by autumn (4.0%±1.6 decade^-1^), winter (1.9%±0.9 decade^-1^) and spring (1.7%±1.2 decade^-1^). We note that the absolute trend values for IOA are relatively small in relation to the size of one grid-cell: ~25 km x ~25 km = 625 km^2^; hence, the annual trend of 3.0x10^3^ km^2^ corresponds to 4–5 grid cells. Overall, we find an increasing trend of sea-ice concentration and extent in the IOA, which is associated with sea surface cooling (Figs 1 and 2).

**Table 1:** Annual and seasonal trends (with standard errors) of sea-ice extent computed for the Antarctica and individual sectors over the period 1979–2015.

Region	Annual		Summer (JFM)		Autumn (AMJ)		Winter (JAS)		Spring (OND)	
	10^3^ km^2^ yr^–1^	% decade^–1^	10^3^ km^2^ yr^–1^	% decade^–1^	10^3^ km^2^ yr^–1^	% decade^–1^	10^3^ km^2^ yr^–1^	% decade^–1^	10^3^ km^2^ yr^–1^	% decade^–1^
SH	24.9±4.4	2.1±0.4	23.6±7.5	5.5±1.7	29.5±7.4	2.7±0.7	19.6±4.3	1.1±0.2	26.8±7.5	1.7±0.5
WS	5.2±1.8	2.6±0.9	20.4±5.4	5.4±3.2	-12.4±9.5	-3.3±2.5	-2.5±4.9	-0.4±0.8	1.9±5.3	0.3±0.9
**IO**	**3.0±1.5**	**2.4±1.2**	**3.7±1.4**	**10.6±4.1**	**5.8±2.3**	**4.0±1.6**	**6.3±3.1**	**1.9±0.9**	**5.0±3.4**	**1.7±1.2**
WPO	2.3±1.7	1.9±1.3	3.8±1.8	7.6±3.6	4.8±1.7	4.1±1.5	0.1±2.6	0.0±1.4	2.2±2.2	1.5±1.5
RS	11.1±2.8	3.8±1.0	7.2±4.2	6.3±3.6	13.3±4.0	4.5±1.3	10.2±3.5	2.5±0.9	13.7±4.0	3.8±1.1
BAS	-3.8±-2.0	-2.5±1.4	-11.6±2.0	-17.8±3.1	-6.8±2.8	-5.2±2.1	4.3±3.4	1.9±1.5	-0.9±3.7	-0.5±2.1

Southern Hemisphere (SH), Weddell Sea (WS), Indian Ocean (IO), Western Pacific Ocean (WPO), Ross Sea (RS), Bellingshausen and Amundsen Seas (BAS)

Analysis of sea-ice freeboard flux data during 2002–2017 suggest a slight increase in meridional northward flux from, at gate 1, around zero before 2010 to ~0.5 km^3^/day afterwards, and at gate 2 from ~0.1 km^3^/day to ~0.4 km^3^/day (S2 Fig). At the two southernmost gates (3 and 5) we find a general import of sea ice from the East at gate 5 and a general export of sea ice towards the west at gate 3 –in accordance with the circum-Antarctic easterly winds (S3 and S4 Figs). We cannot find a change in this import and export over the time period considered. The two northernmost gates (4 and 6) do not reveal any trends either (S3 and S4 Figs). At gate 6 flux values are too sparse and suggest a sea-ice important. At gate 4, import and export seem to be in balance–except in the year 2010 where sea ice was imported into the IOA from the Weddell Sea (positive = eastward sea-ice freeboard flux).

What causes the marked increase in regional sea-ice with the background of global warming? Antarctic sea-ice expansion has been attributed to the positive trends in the SAM, associated with the poleward shifting of westerly wind [16] that reduces poleward heat transport and induces cooling of the Antarctic atmosphere [12,14]. However, the magnitude of the Antarctic sea-ice changes related to the SAM and El Niño–Southern Oscillation (ENSO) were found to be smaller than the regional sea-ice trends [7]. Our result shows SAM index (1979–2015) has a significant trend towards high-index polarity during the austral summer and autumn that explains about 14% to 33% variance of the IOA sea-ice extent (S5 Fig and S1 Table). The observed high-index polarity in SAM is influenced largely by the stratospheric ozone depletion over the Antarctica [17,19]; possibly leads to Antarctic sea-ice increase [18,20]. An experiment using atmospheric climate model revealed that the deepening of the Amundsen–Bellingshausen Sea low in response to the stratospheric ozone depletion is accountable to the regional sea-ice variability [18]. It is explained that the development of strong cyclonic circulation induced by stratospheric ozone depletion intensifies windspeeds in austral autumn, deepens the Amundsen Sea Low, which plays a key role in regional sea-ice expansion. In contrast, studies using coupled climate models showed that ozone depletion over the Antarctica supposed to result the Southern Ocean warming, and sea-ice loss, did not contribute significantly to the observed sea-ice expansion [21,28,47–49]. We find the ozone depletion explains only about 17% of variance in the IOA sea-ice extent during the austral summer and no significant correlation found in other seasons (S1 Table).

Although numerous studies were carried out to understand the atmospheric forcing on recent Antarctic sea-ice trend, the role of oceanic influence on sea-ice has become difficult to carry out due to the scarcity of *in-situ* data [18]. Regional studies over the Antarctica have shown accelerated freshening due to the increased precipitation and melting of the west Antarctic ice-sheet that enhances the thermohaline stratification and weakens the convective overturning, in turn reduces the ocean heat flux to, on the one hand, melt sea-ice and other hand promote more sea-ice formation [50,51]. The oceanic heat flux available for sea-ice melting decreases faster than the sea-ice growth in the weakly stratified Southern Ocean that favours sea-ice expansion [4]. Another mechanism suggested that increased freshwater input by and mass loss of the Antarctic ice sheet are caused by subsurface ocean warming which can drive enhanced basal melting of ice-shelves [23,52]. However, the gravimetric mass balance analysis of the ice-sheets adjacent to the IOA revealed a gain of about 6.83±0.5 Gt of mass annually (S6 Fig). With this background, the following possible mechanism is proposed to explain the observed sea-ice expansion in the IOA.

### Possible mechanism of sea-ice expansion

The basal and upper portions of the Antarctica ice shelves are vulnerable to changes in ocean and atmospheric forcing, respectively. Indeed, the melting rates are faster owing to the subsurface warming of the Southern Ocean [23,25,53–57]. The melt water accumulates as a cool and fresh layer, thereby enhances the vertical stability and promoting sea-ice expansion [23]. In the Eastern Antarctica, the ice-sheets are gaining mass due to the enhanced precipitation [58] (S6 Fig) and eventually the ice shelves melt either locally through basal melting or remotely by drifting of icebergs [56]. In either case of mass gain or loss, the drainage networks are expected to remain active and discharges more fresh water from the ice shelves into the ocean during each austral summer, in addition to contribution of freshwater input from sea-ice melting. The persistent drainage network, interconnected streams, ponds and rivers of the Antarctica export large amount of the ice shelves melt water (fresh water) into the ocean through waterfalls and dolines [59]. The melt water spreads and stratifies as low saline, cold water at the upper ocean due to its lower density because the salinity dominates the density structure in the Southern Ocean [60].

**Fig 3:**
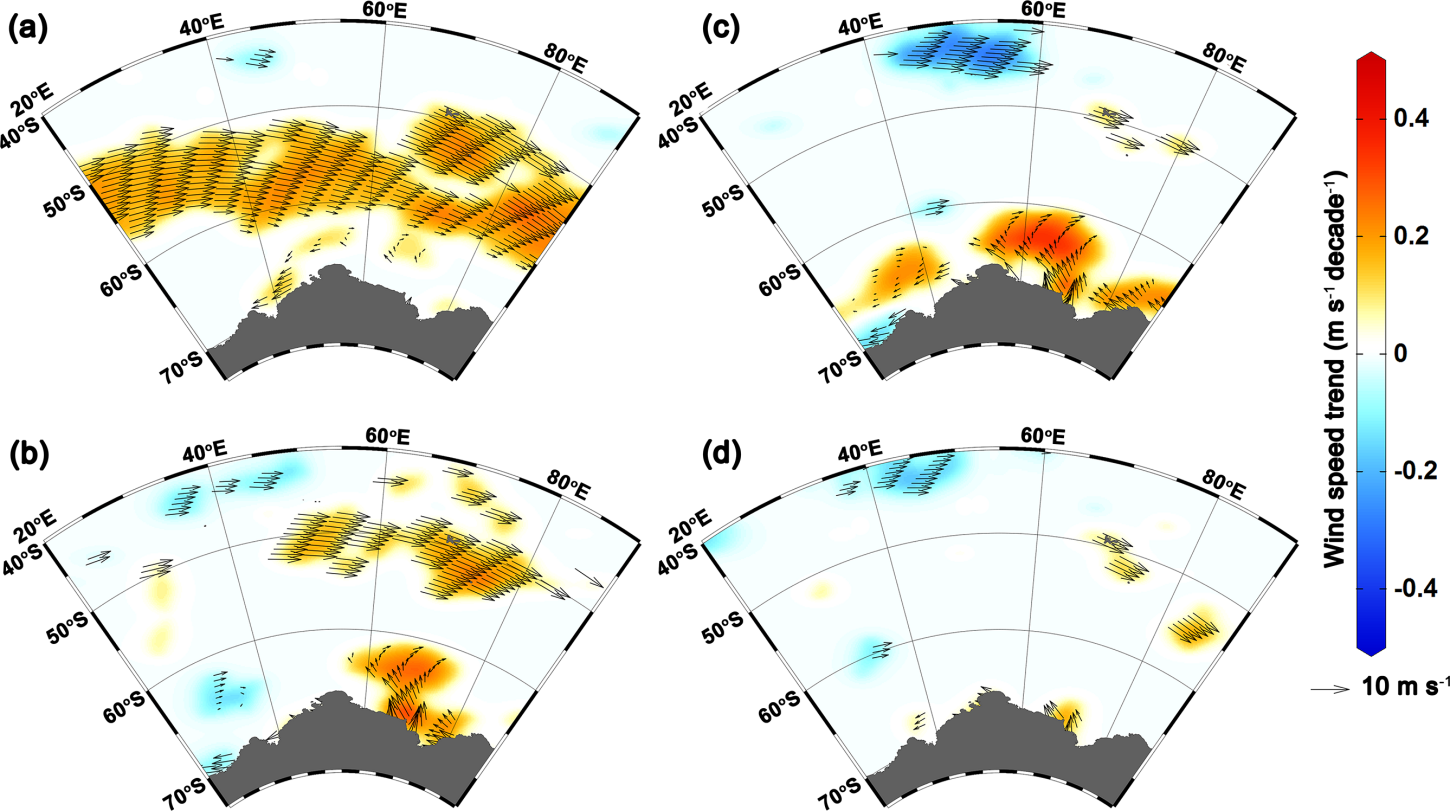
Spatial distribution of ERA-Interim 10m-wind speed trend (m s^-1^ decade^-1^) at p<0.05, with the vectors showing the seasonal climatology for (**a**) summer, (**b**) autumn, (**c**) winter, and (**d**) spring, over the period of 1979–2015.

**Fig 4:**
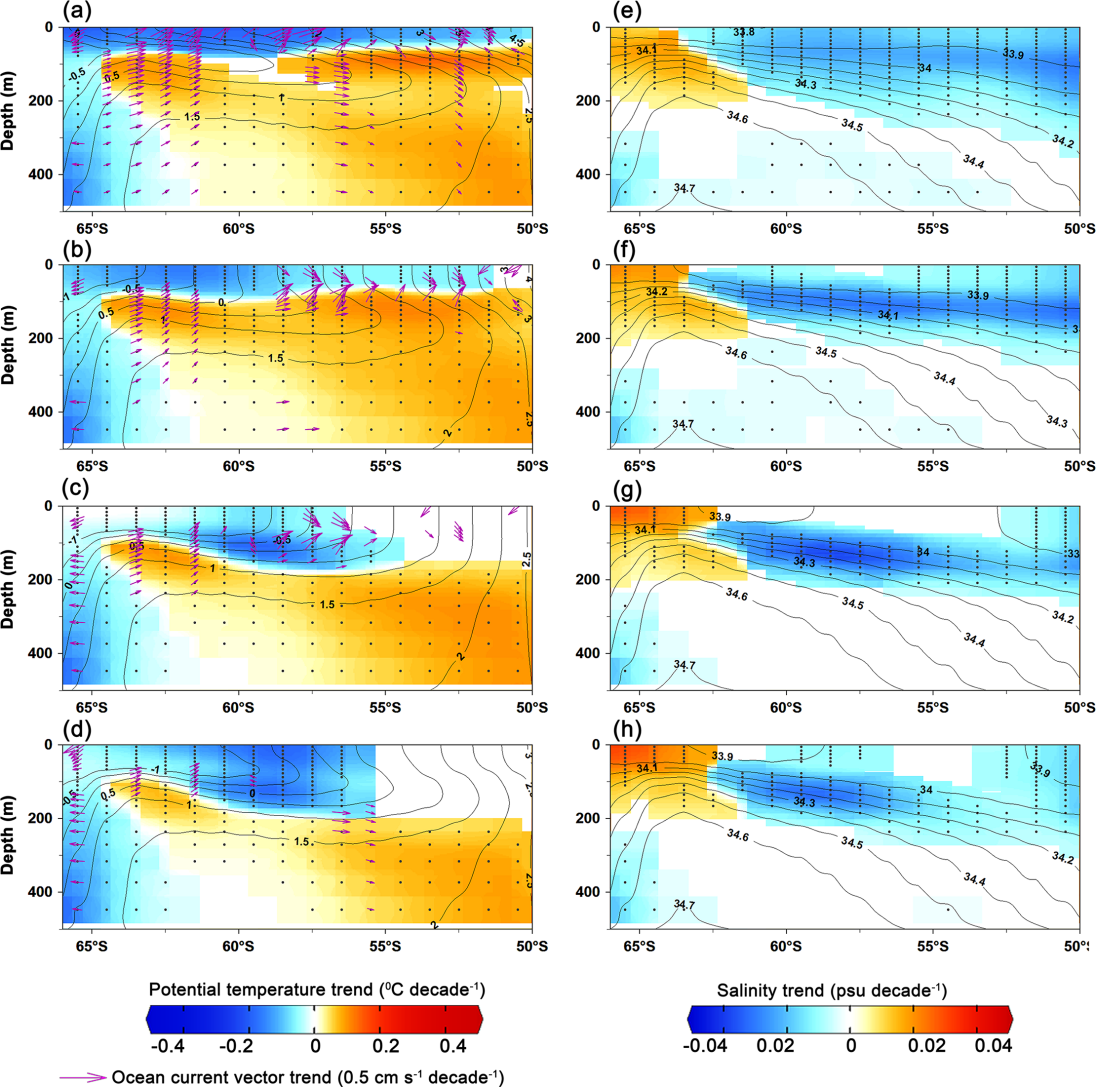
Depth-Latitude cross section of zonally averaged (20°E to 90°E) trends of potential temperature for (**a**) summer, (**b**) autumn, (**c**) winter, and (**d**) spring. The trends of ocean current vectors are overlaid as magenta arrows (in figures a-d). Right panel figures indicate the trends of salinity for (**e**) summer, (**f**) autumn, (**g**) winter, and (**h**) spring. Only the significant trend values (*p* < 0.05) are considered to plot the figures according to a two-tailed t-test and the dots are marked to show the observations with the significant trends. The contour shows the climatology of temperature (left panel) and salinity (right panel). The data are obtained from the European Centre for Medium-Range Weather Forecast (ECMWF)’s Ocean Reanalysis System 4 (ORAS4) for the period of 1979–2015.

**Fig 5:**
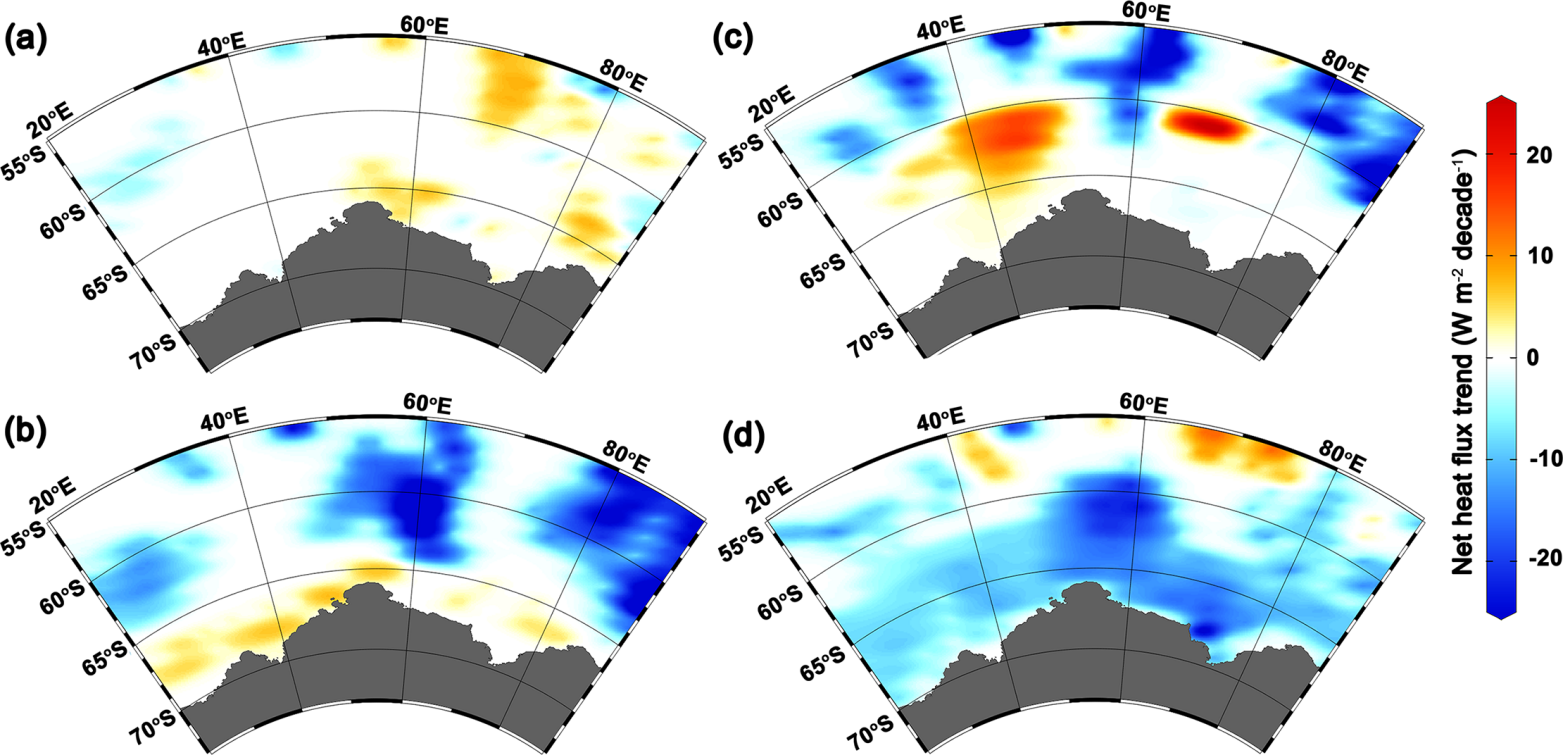
Spatial distribution of net heat flux trend (W m^-2^ decade^-1^) at p<0.05, over the period of 1979–2011, for (**a**) summer, (**b**) autumn, (**c**) winter, and (**d**) spring. Positive values indicate heat gain by the sea surface and negative values indicate heat loss from the sea surface. Significant heat loss trend observed in larger portion of the Indian Ocean sector of Antarctica (IOA) particularly in austral autumn, winter and spring.

**Fig 6:**
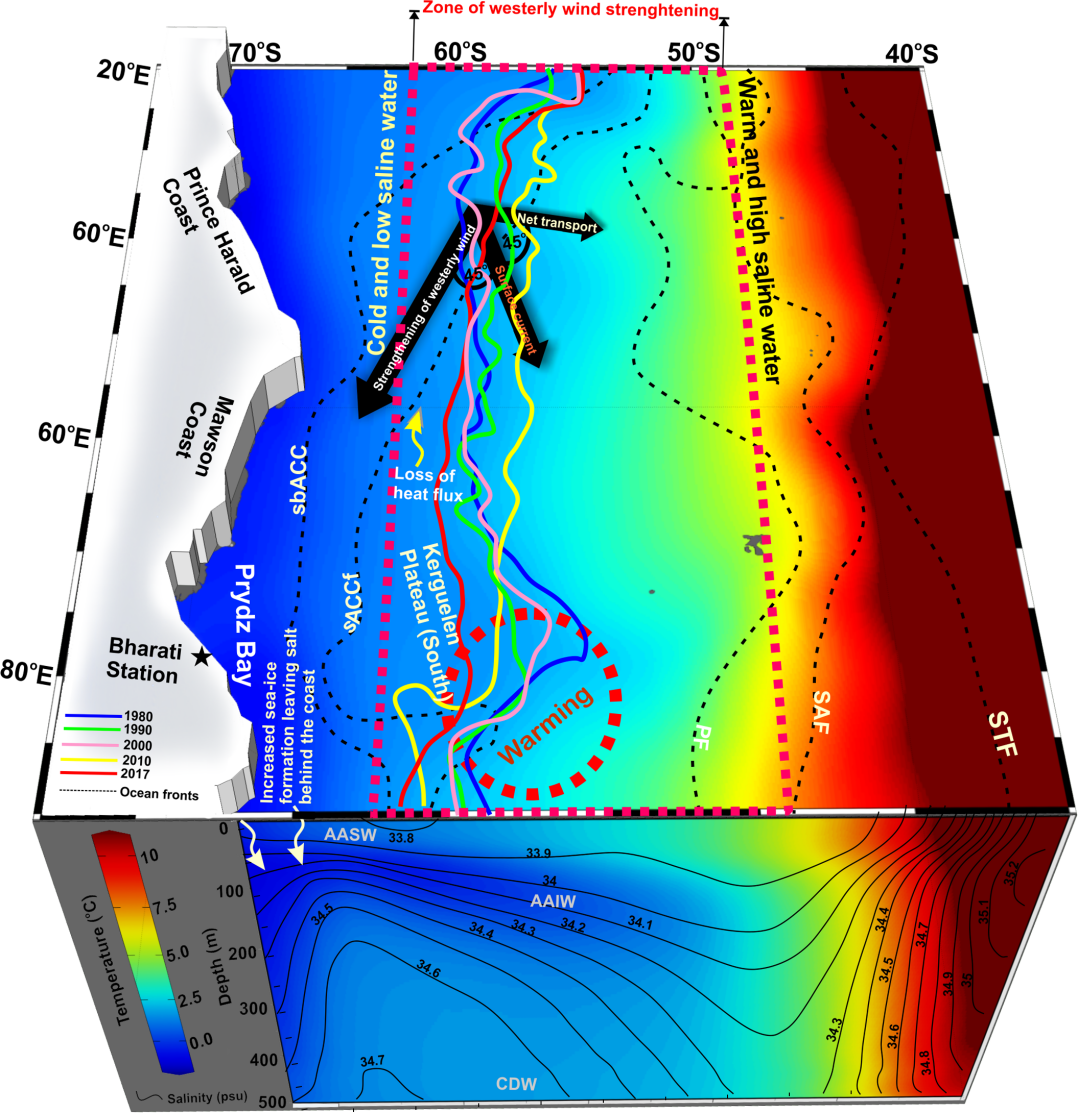
Schematic diagram illustrates the mechanism of sea-ice expansion in the Indian Ocean sector of Antarctica (IOA). The background colour shows the mean state of sea surface temperature along with the cross section of zonally averaged (20°E to 90°E) temperature and salinity (contours) up to 500m depth, utilizing Ocean Reanalysis System 4 (ORAS4) for the period of 1979–2015. Dashed rectangle (magenta) shows the zone of westerly wind strengthening in austral summer suggests northward advection (blue-red blended arrow) of low saline and cool water from the southern boundary of the Antarctic circumpolar current (sbACC), accompanied with the upper ocean cooling through an overall loss of net heat flux (black spiral arrow), thus favours overall sea-ice expansion. Dashed circle (red) shows the region of consistent warming trend in the entire water column on the south Kerguelen Plateau, lead to the localized sea-ice retreat. Solid lines (colour) show the sea-ice extent in September with an increasing pattern over the larger portion of IOA, except on the south Kerguelen Plateau. A notable episodic event on sea-ice retreat during 2017 is linked to the enhanced poleward advection of warm and moist air that led to strong melting [69]. Locations of climatological Southern Ocean fronts are represented as black dashed lines [32]. White arrows show salinification near the coast (south of ~62°S) due to enhanced rejection of salt associated with high rate of sea-ice production and its export [23,65].

Our analysis reveals significant strengthening of westerly wind (up to 0.33±0.07 m s^-1^ decade^-1^) and thereby increasing the surface current (up to 0.35±0.08 cm s^-1^ decade^-1^) in the austral summer (between 50°S to 62°S) suggesting northward transport of these comparably fresh, cold water from the southern boundary of the Antarctic circumpolar current (Figs 3 and 4A–4D). The strengthening of westerly wind is driven by the tropospheric pressure variability, which has oscillated southward (south of 60°S) and northward between 30°S to 50°S [13,20]. The strengthening of westerly wind facilitates an anomalous sea surface cooling and freshening north of 62°S, due to northward movement of cold fresh water and net heat loss, during the austral summer that persists in the successive seasons and favours for sea-ice expansion in the IOA (Fig 4). A slight increase in northward flux of sea-ice freeboard is also evident in S2 Fig. This finding is supported by the ORAS4 and EN4, showing widespread freshening rate up to -0.02 ±0.004 psu decade^-1^ in the surface and intermediate waters north of 62°S (Fig 4E–4H, S7 Fig), which is consistent with the earlier observations on freshening of comparable magnitude [61–63]. The observed freshening has been reported in different sector of the Southern Ocean, attributed to the accelerated freshwater fluxes from the Antarctic glacial melt [50,64], atmospheric fluxes by excess precipitation over evaporation [62], and the northward transport of sea-ice [9,63,65]. The accelerated freshening of sea surface can lower the SST by weakening convection and vertical mixing through enhanced thermohaline stratification in the water column, which favours the sea-ice expansion [4]. The widespread sea surface cooling up to -0.2±0.01°C decade^-1^ observed in all seasons in synchronise with sea-ice expansion in the IOA, except few contrasting features at some places near to the coast and south Kerguelen Plateau (Fig 1). The cooling trend prevails in the upper layer, whereas the subsurface warming is observed below ~100 m depth (Fig 4A–4D). The subsurface warming is possibly due to the advection of circumpolar deep water (CDW) which delivers warm water onto the ice shelves through submarine troughs and leads to basal melting [24,57,66,67]. The subsurface warming and its effect on ice shelves basal melting in turn helps accumulation of low saline and cold water at the sea surface [23]. The observed strengthening of the westerly wind in austral summer induces the northward transport of these low saline, cool water that freezes readily into a crystalline structure of sea-ice compared to the displaced water of relatively high saline and warm waters. Concurrently, the westerly wind in austral summer generates the robust feature of upper ocean cooling in the successive seasons through an overall loss of net heat flux from the sea surface (Figs 2 and 5, S8 Fig). The joint impact of these processes produces anomalous upper ocean cooling and freshening north of 62°S, persists in the successive seasons and possibly explains the observed sea-ice expansion (autumn and winter) while the sea-ice forms and grows (Fig 6). However, salinification is observed near the coast (south of ~62°S) due to enhanced rejection of salt associated with high rate of sea-ice production and its export [23,63,65]. This salinification across the coast in coastal polynyas by sea-ice growth has interesting implications—partly for the sea-ice cover itself, via ice production and export of sea-ice out of, e.g. the Cape Darnley polynya and Prydz Bay polynyas as well as smaller polynyas further west, but in particular also in terms of water mass modification and anticipated dense water mass production [68].

The dominance of increasing sea-ice concentration and decreasing SST observed near the marginal ice zone, where sea-ice growth is evident (Fig 1 and S9 Fig). Interestingly, an exception is observed on the reduction of sea-ice concentration near the south Kerguelen Plateau and coastal regions of the Prydz Bay, Mawson and Prince Harald, triggered by enhanced warming throughout the water column both at the surface and subsurface (Fig 1 and S10 Fig). In particular, the waters above the southern Kerguelen Plateau (55°S to 60°S and 80°E to 90°E) exhibit a consistent warming trend up to 0.26±0.01°C decade^-1^ with a concurrent reduction in sea-ice concentration up to 2% decade^-1^. Retreat in sea-ice extent is recorded on the south Kerguelen Plateau, showing a non-annular pattern of sea-ice variability in the IOA (Fig 6).

## Conclusions

This study reveals significant sea-ice expansion in the IOA associated with the robust feature of sea surface cooling trend. SAM and stratospheric ozone depletion over the Antarctica partially explained the variability in the IOA sea-ice extent. We suggest a mechanism which shows significant strengthening of the westerly wind in austral summer that induces northward transport of the low saline and cold water, accompanied with the upper ocean cooling through an overall loss of net heat flux, conducive for sea-ice expansion. Apparently, an increasing pattern of sea-ice concentration and a decrease in SST, are maximum near the marginal ice zone. Global ocean reanalysis data support our conclusion, showing widespread freshening at the surface and intermediate waters. The upper ocean (~100 m) cooled significantly, except the warming and corresponding sea-ice reduction near the south Kerguelen Plateau, and coastal regions of the Prydz Bay, Mawson, Prince Harald. The remainder water column below ~100 m warmed significantly, which has been detected earlier and linked to the advection of CDW that delivers warm water onto the ice shelves [24,57,66]. In the coming decades, a subsurface warming of the Southern Ocean may be favourable for sea-ice expansion through ice shelves basal melting through accumulation of the low saline and cool water at the sea surface [23]. Despite the fact of Antarctic sea-ice expansion during 1979–2015, the record rate of springtime sea-ice reduction in 2016–2017 is a further challenge to our understanding on the Antarctic sea ice variability in a moderately short satellite observational record [69] (Fig 6). This episodic sea-ice reduction has been ascribed to the persistence of the negative index polarity of SAM in springtime led to westerly wind weakening and an overall surface warming of the coastal Antarctica. The overall warming is linked to the enhanced poleward advection of warm and moist air led to strong sea-ice melting. Regional atmosphere-ocean-sea-ice modelling is imperative to characterise the sea-ice dynamics and quantify the individual contribution of various physical processes. This study describes one of the realistic possible mechanisms that explain the overall increasing trend of satellite derived sea-ice in the IOA.

## Supporting information

S1 Table Cross-correlation (*r*) between sea-ice extent and ocean-atmospheric parameters for different seasons with the significant relationship (*p* < 0.05) marked in bold. (DOCX)

S1 Fig Location of flux gates superposed onto a map of the sea-ice freeboard distribution. Sea-ice freeboard fluxes are computed across 64°S latitude (gates 1 and 2), at the western (gates 3 and 4 at 20°E) and eastern (gates 5 and 6 at 100°E) entry to the Indian Ocean sector. The red line at ~53°E separates between western IO = Weddell East and eastern IO = East Antarctic West.
(TIF)

S2 Fig Monthly total sea-ice freeboard flux across gates 1 and 2 (see S1 Fig). Years and vertical dashed lines denote the beginning of the respective calendar year.
(TIF)

S3 Fig Monthly total sea-ice freeboard flux across gates 3 and 4, i.e. the western entry to our region of interest (see S1 Fig). (TIF)

S4 Fig Monthly total sea-ice freeboard flux across gates 5 and 6, i.e. the eastern entry to our region of interest (see S1 Fig). (TIF)

S5 Fig Time series of seasonal mean SAM index (Marshall., 2003) over the period of 1979–2015. The dashed lines are the running mean of 10-year data for different seasons; indicates significant trend towards high-index polarity during the austral summer (dashed red line) and autumn (dashed black line).
(TIF)

S6 Fig Annual mass variability of the Antarctic ice-sheets adjacent to the Indian Ocean sector of Antarctica, showed a gain of about 6.83±0.5 Gt yr^-1^. The data are obtained from the Technische Universität Dresden, Germany, derived from the Gravity Recovery and Climate Experiment (GRACE) satellite.
(TIF)

S7 Fig The European Centre for Medium range Weather Forecast (ECMWF)’s EN4 data shows the cross section of the zonally averaged (20°E to 90°E) trends of potential temperature for (**a**) summer, (**b**) autumn, (**c**) winter, and (**d**) spring, over the period of 1979–2015. Right panel figures indicates the trends of salinity for (**e**) summer, (**f**) autumn, (**g**) winter, and (**h**) spring. Only the significant trend values (*p* < 0.05) are considered to plot the figures according to a two tailed t-test and the dots are marked to show the observations with the significant trends. The contours indicate the climatology of temperature and salinity overlaid on the color shaded trend maps.
(TIF)

S8 Fig Sea surface temperature (SST) anomaly computed from multiple data showed robust feature of sea surface cooling over the Indian Ocean sector of Antarctica (averaged from 20°E to 90°E and 73°S to 55°S), for (**a**) summer, (**b**) autumn, (**c**) winter, and (**d**) spring. The optimum interpolated SST (OI SST), Interim SST, Ocean Reanalysis System 4 (ORAS4) SST and EN4 SST data are used for the analysis. EN4 SST (pink line) shows large deviations (probably uncertainties) compared to other datasets, particularly during austral autumn and winter.
(TIF)

S9 Fig Sea-ice concentration trend contours (% decade^-1^) overlaid on the sea-ice concentration climatology (1979–2015), for (**a**) summer, (**b**) autumn, (**c**) winter, and (**d**) spring, generated using ERA-Interim reanalysis data. Only the significant trend values (*p* < 0.05) are contoured according to a two tailed t-test. Overall, the increasing pattern of sea-ice concentration is evident in the Indian Ocean sector of the Southern Ocean except the reduction in sea-ice near the south Kerguelen Plateau (SKP), and coastal regions of the Prydz Bay (PB), Mawson (MS), Prince Harald (PH).
(TIF)

S10 Fig The cross section of the zonal temperature trend at 85°E (south Kerguelen Plateau) for (**a**) summer, (**b**) autumn, (**c**) winter, and (**d**) spring, over the period of 1979–2015, computed from the European Centre for Medium range Weather Forecast (ECMWF)’s Ocean Reanalysis System 4 (ORAS4) data. Enhanced warming is observed throughout the water column both at the surface and subsurface with a concurrent reduction in sea-ice as shown in Figs 2 and 6.
(TIF)
