# Supplementary material for: Mechanism of sea-ice expansion in the Indian Ocean sector of Antarctica: Insights from satellite observation and model reanalysis
Source: PLoS One. 2018 Oct 3;13(10):e0203222. doi: 10.1371/journal.pone.0203222 (PMC6169864; doi:10.1371/journal.pone.0203222)
Supplement: S1 Table — (DOCX) [file pone.0203222.s001.docx]

**Table ST1.**

| Parameters | Sea-ice extent | OI SST | Wind speed | Zonal wind | Meridional wind | Net heat flux | SAM | Total columnozone |  |
| --- | --- | --- | --- | --- | --- | --- | --- | --- | --- |
| **Summer** | | | | | | | | | |
| Sea-ice extent | 1.00 |  |  |  |  |  |  |  |  |
| OI SST | **-0.76** | 1.00 |  |  |  |  |  |  |  |
| Wind speed | **0.40** | **-0.44** | 1.00 |  |  |  |  |  |  |
| Zonal wind | **0.40** | **-0.37** | **0.56** | 1.00 |  |  |  |  |  |
| Meridional wind | -0.16 | 0.32 | **-0.45** | **-0.48** | 1.00 |  |  |  |  |
| Net heat flux | 0.18 | -0.12 | -0.11 | -0.29 | 0.27 | 1.00 |  |  |  |
| SAM | **0.38** | -0.26 | **0.51** | **0.79** | -0.31 | -0.20 | 1.00 |  |  |
| Total ozone column | **-0.41** | **0.39** | **-0.39** | -0.17 | 0.17 | -0.25 | **-0.40** | 1.00 |  |
| **Autumn** | | | | | | | | | |
| Sea-ice extent | 1.00 |  |  |  |  |  |  |  |  |
| OI SST | **-0.71** | 1.00 |  |  |  |  |  |  |  |
| Wind speed | 0.27 | -0.25 | 1.00 |  |  |  |  |  |  |
| Zonal wind | **0.34** | -0.25 | **0.51** | 1.00 |  |  |  |  |  |
| Meridional wind | 0.26 | -0.03 | -0.25 | **-0.41** | 1.00 |  |  |  |  |
| Net heat flux | -0.21 | 0.28 | -0.10 | 0.30 | **-0.34** | 1.00 |  |  |  |
| SAM | **0.58** | **-0.37** | **0.50** | **0.78** | -0.11 | -0.09 | 1.00 |  |  |
| Total ozone column | -0.15 | 0.19 | -0.12 | 0.08 | -0.25 | 0.04 | 0.08 | 1.00 |  |
| **Winter** | | | | | | | | | |
| Sea-ice extent | 1.00 |  |  |  |  |  |  |  |  |
| OI SST | **-0.86** | 1.00 |  |  |  |  |  |  |  |
| Wind speed | 0.09 | -0.12 | 1.00 |  |  |  |  |  |  |
| Zonal wind | 0.13 | -0.09 | **0.76** | 1.00 |  |  |  |  |  |
| Meridional wind | 0.02 | 0.11 | **-0.49** | **-0.64** | 1.00 |  |  |  |  |
| Net heat flux | -0.30 | **0.44** | 0.30 | **0.51** | -0.11 | 1.00 |  |  |  |
| SAM | 0.28 | -0.21 | **0.74** | **0.87** | **-0.50** | **0.38** | 1.00 |  |  |
| Total ozone column | -0.07 | 0.06 | -0.09 | -0.03 | -0.06 | 0.05 | -0.16 | 1.00 |  |
| **Spring** | | | | | | | | | |
| Sea-ice extent | 1.00 |  |  |  |  |  |  |  |  |
| OI SST | **-0.65** | 1.00 |  |  |  |  |  |  |  |
| Wind speed | -0.02 | 0.01 | 1.00 |  |  |  |  |  |  |
| Zonal wind | 0.14 | -0.26 | **0.46** | 1.00 |  |  |  |  |  |
| Meridional wind | -0.30 | **0.36** | **-0.43** | **-0.67** | 1.00 |  |  |  |  |
| Net heat flux | **-0.56** | **0.83** | -0.01 | -0.23 | 0.19 | 1.00 |  |  |  |
| SAM | **0.42** | **-0.65** | 0.19 | **0.48** | -0.25 | **-0.49** | 1.00 |  |  |
| Total ozone column | -0.25 | **0.33** | **-0.55** | -0.28 | 0.27 | **0.40** | -0.32 | 1.00 |  |
