# Supplementary figures and images for: Mechanism of sea-ice expansion in the Indian Ocean sector of Antarctica: Insights from satellite observation and model reanalysis
Source: PLoS One. 2018 Oct 3;13(10):e0203222. doi: 10.1371/journal.pone.0203222 (PMC6169864; doi:10.1371/journal.pone.0203222)

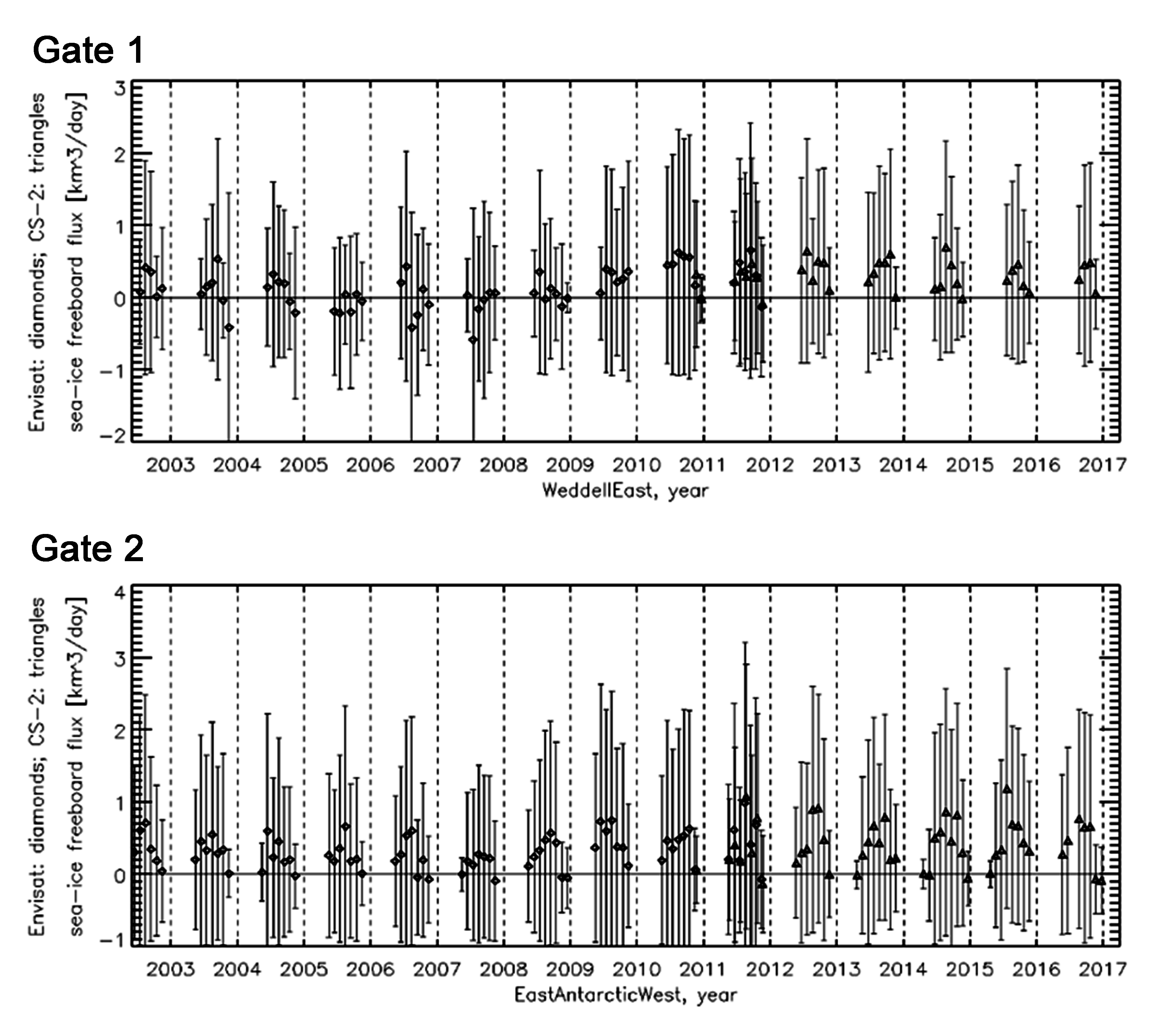

Supplement: S2 Fig — Years and vertical dashed lines denote the beginning of the respective calendar year. (TIF) [file pone.0203222.s003.tif]

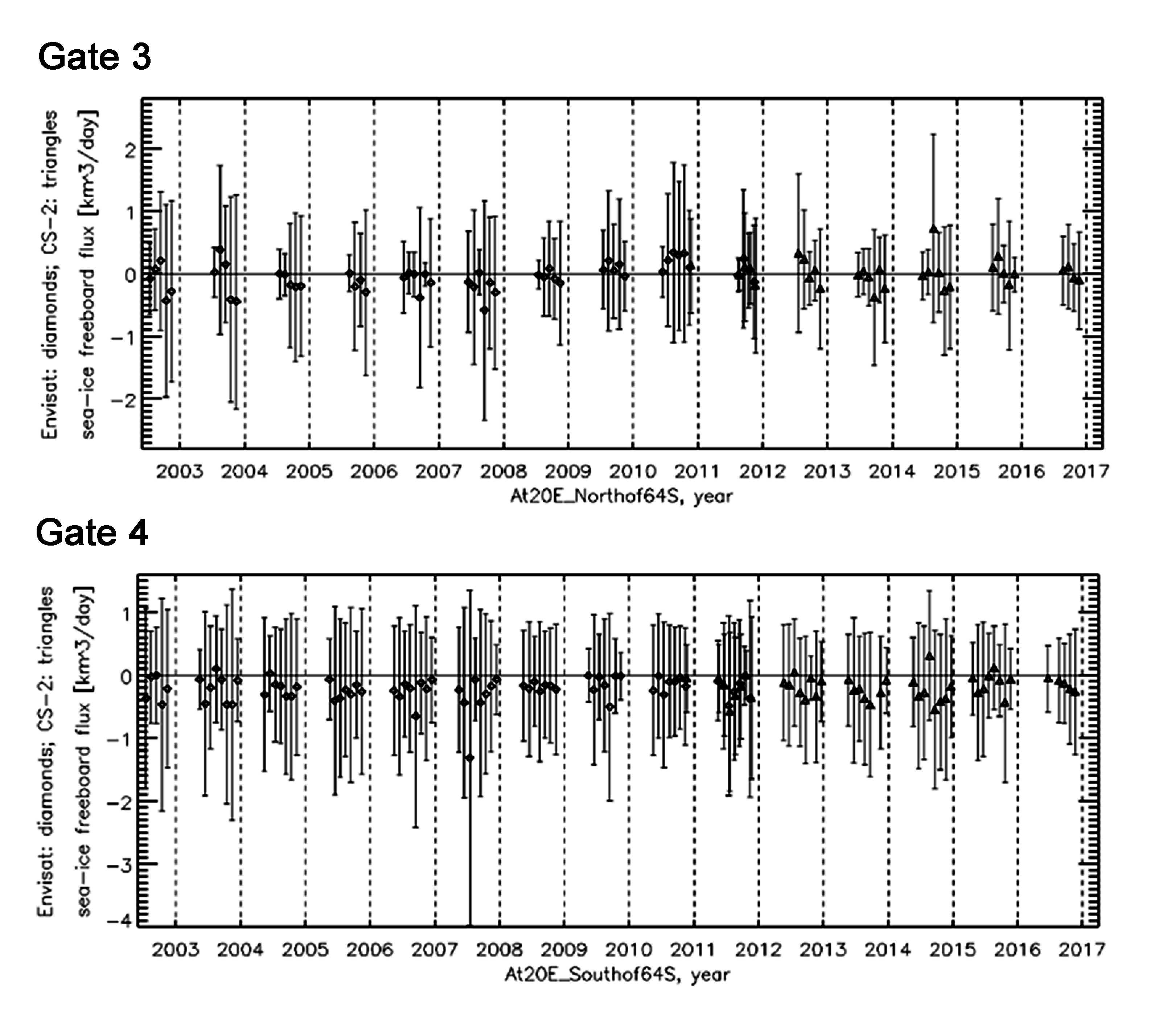

Supplement: S3 Fig — (TIF) [file pone.0203222.s004.tif]

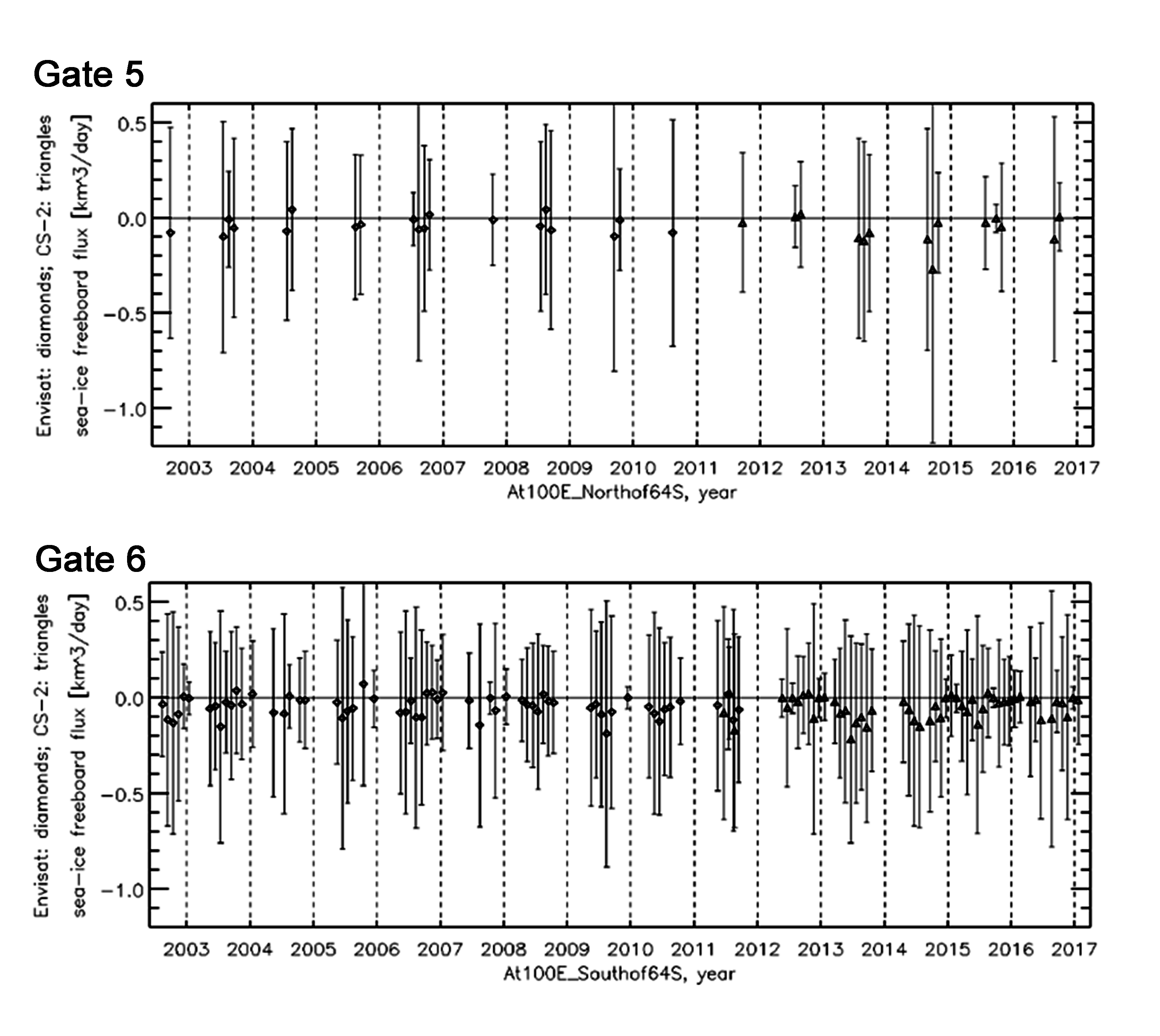

Supplement: S4 Fig — (TIF) [file pone.0203222.s005.tif]

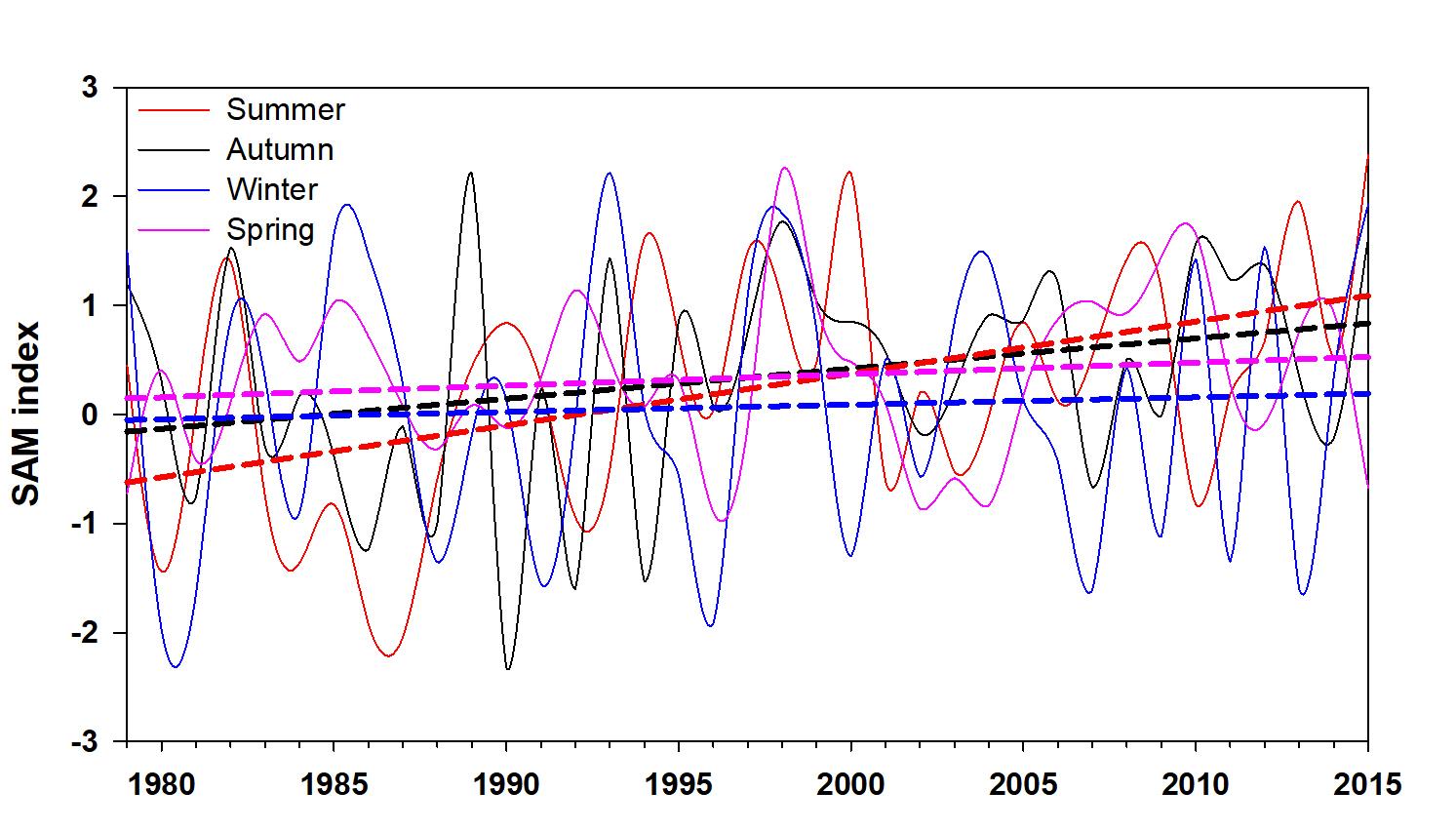

Supplement: S5 Fig — The dashed lines are the running mean of 10-year data for different seasons; indicates significant trend towards high-index polarity during the austral summer (dashed red line) and autumn (dashed black line). (TIF) [file pone.0203222.s006.tif]

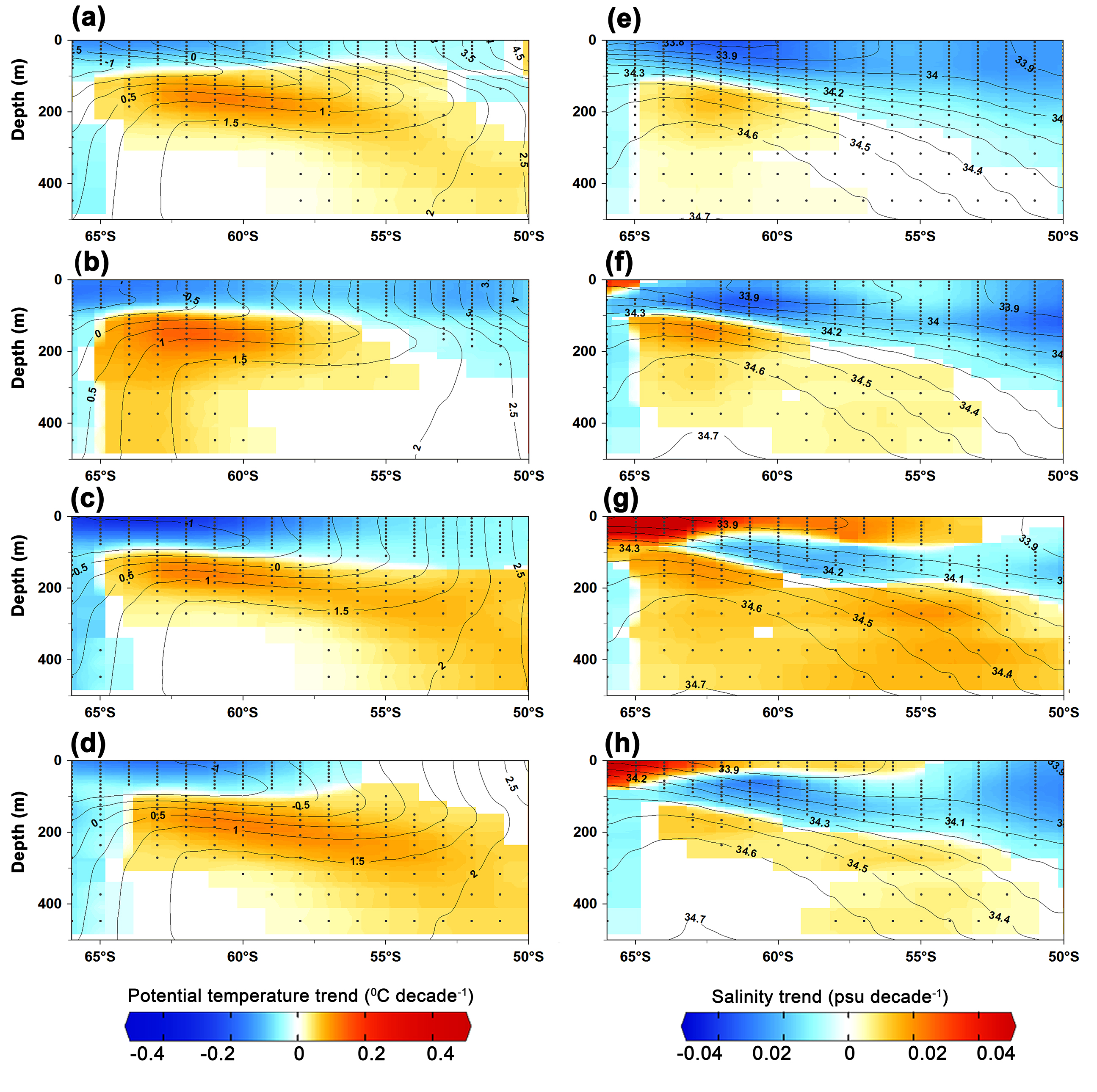

Supplement: S7 Fig — The European Centre for Medium range Weather Forecast (ECMWF)’s EN4 data shows the cross section of the zonally averaged (20°E to 90°E) trends of potential temperature for (a) summer, (b) autumn, (c) winter, and (d) spring, over the period of 1979–2015. Right panel figures indicates the trends of salinity for (e) summer, (f) autumn, (g) winter, and (h) spring. Only the significant trend values (p < 0.05) are considered to plot the figures according to a two tailed t-test and the dots are marked to show the observations with the significant trends. The contours indicate the climatology of temperature and salinity overlaid on the color shaded trend maps. (TIF) [file pone.0203222.s008.tif]

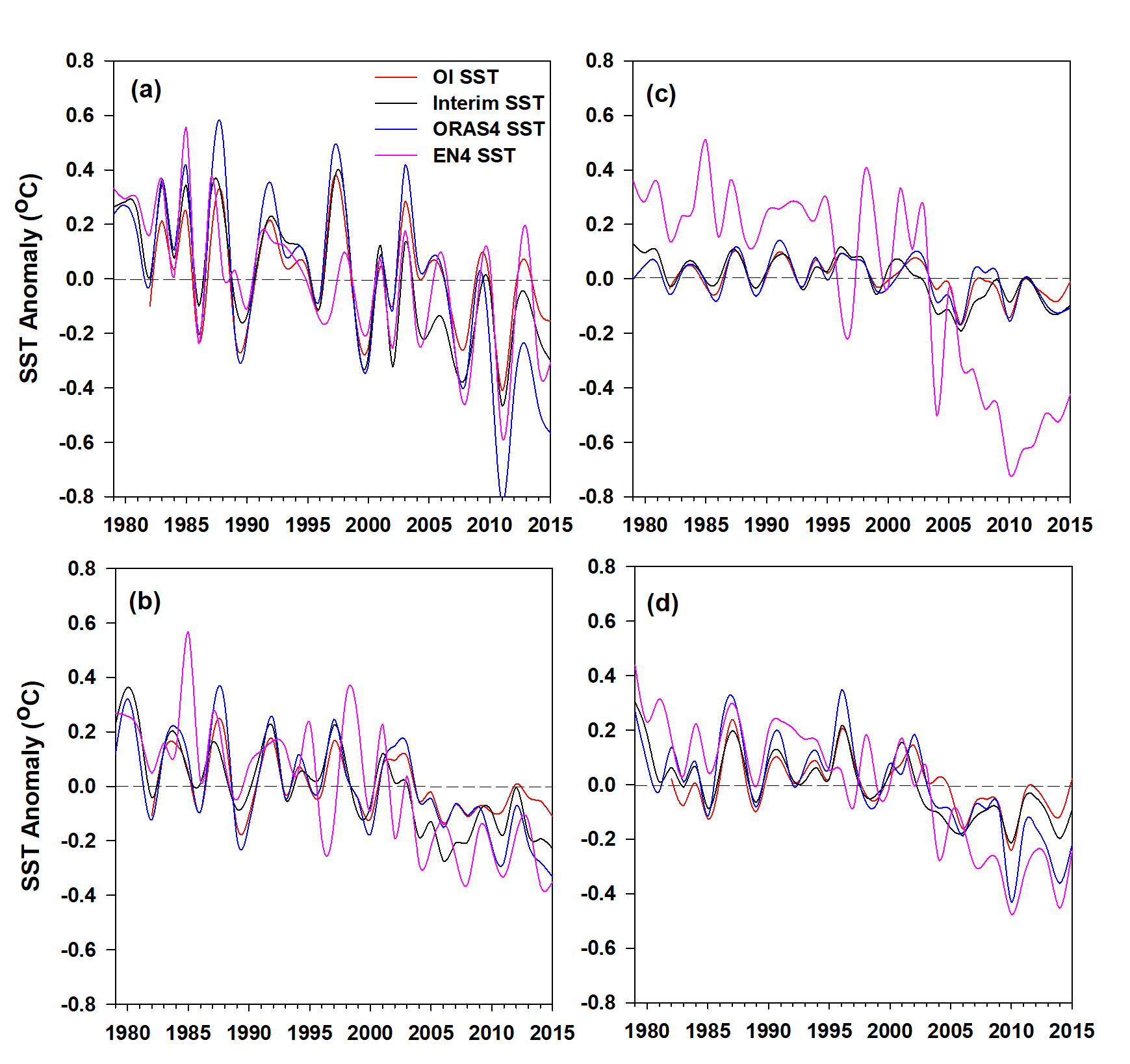

Supplement: S8 Fig — Sea surface temperature (SST) anomaly computed from multiple data showed robust feature of sea surface cooling over the Indian Ocean sector of Antarctica (averaged from 20°E to 90°E and 73°S to 55°S), for (a) summer, (b) autumn, (c) winter, and (d) spring. The optimum interpolated SST (OI SST), Interim SST, Ocean Reanalysis System 4 (ORAS4) SST and EN4 SST data are used for the analysis. EN4 SST (pink line) shows large deviations (probably uncertainties) compared to other datasets, particularly during austral autumn and winter. (TIF) [file pone.0203222.s009.tif]

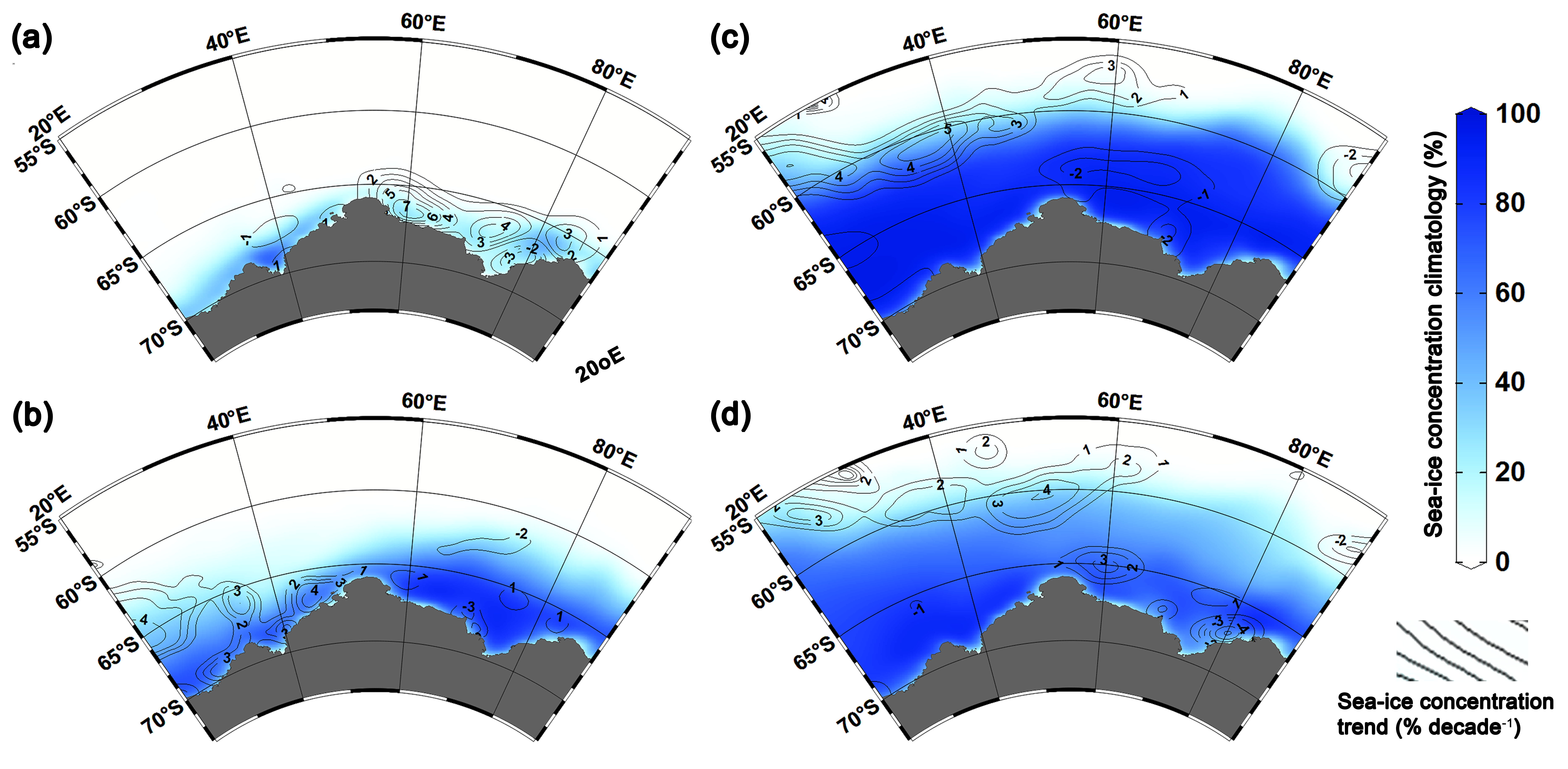

Supplement: S9 Fig — Sea-ice concentration trend contours (% decade-1) overlaid on the sea-ice concentration climatology (1979–2015), for (a) summer, (b) autumn, (c) winter, and (d) spring, generated using ERA-Interim reanalysis data. Only the significant trend values (p < 0.05) are contoured according to a two tailed t-test. Overall, the increasing pattern of sea-ice concentration is evident in the Indian Ocean sector of the Southern Ocean except the reduction in sea-ice near the south Kerguelen Plateau (SKP), and coastal regions of the Prydz Bay (PB), Mawson (MS), Prince Harald (PH). (TIF) [file pone.0203222.s010.tif]

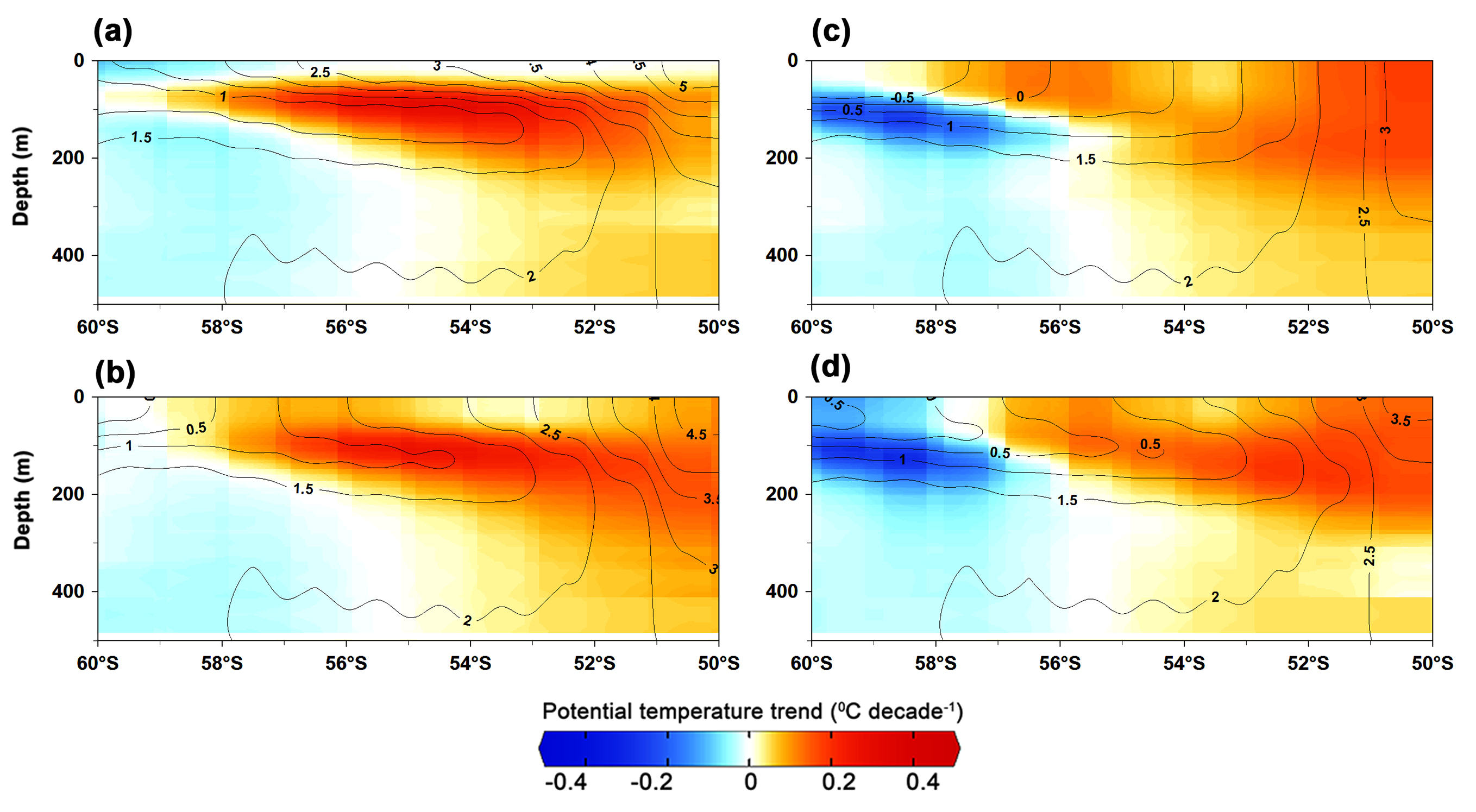

Supplement: S10 Fig — The cross section of the zonal temperature trend at 85°E (south Kerguelen Plateau) for (a) summer, (b) autumn, (c) winter, and (d) spring, over the period of 1979–2015, computed from the European Centre for Medium range Weather Forecast (ECMWF)’s Ocean Reanalysis System 4 (ORAS4) data. Enhanced warming is observed throughout the water column both at the surface and subsurface with a concurrent reduction in sea-ice as shown in Figs 2 and 6. (TIF) [file pone.0203222.s011.tif]
